# Epigenetic modifications and their relation to caste and sex determination and adult division of labor in the stingless bee *Melipona scutellaris*

**DOI:** 10.1590/1678-4685-GMB-2016-0242

**Published:** 2017-03-02

**Authors:** Carlos A.M. Cardoso-Júnior, Patrícia Tieme Fujimura, Célio Dias Santos-Júnior, Naiara Araújo Borges, Carlos Ueira-Vieira, Klaus Hartfelder, Luiz Ricardo Goulart, Ana Maria Bonetti

**Affiliations:** 1 Departamento de Genética e Bioquímica, Universidade Federal de Uberlândia, Uberlândia, MG, Brazil.; 2 Departamento de Biologia Celular e Molecular e Bioagentes Patogênicos, Faculdade de Medicina de Ribeirão Preto, Universidade de São Paulo, Ribeirão Preto, SP, Brazil.; 3 Departmento de Genética e Evolução, Universidade Federal de São Carlos, São Carlos, SP, Brazil.

**Keywords:** DNA methylation, histone modification, caste development, social bees, genetic caste determination

## Abstract

Stingless bees of the genus *Melipona*, have long been considered an enigmatic case among social insects for their mode of caste determination, where in addition to larval food type and quantity, the genotype also has a saying, as proposed over 50 years ago by Warwick E. Kerr. Several attempts have since tried to test his Mendelian two-loci/two-alleles segregation hypothesis, but only recently a single gene crucial for sex determination in bees was evidenced to be sex-specifically spliced and also caste-specifically expressed in a *Melipona* species. Since alternative splicing is frequently associated with epigenetic marks, and the epigenetic status plays a major role in setting the caste phenotype in the honey bee, we investigated here epigenetic chromatin modification in the stingless bee *Melipona scutellaris*. We used an ELISA-based methodology to quantify global methylation status and western blot assays to reveal histone modifications. The results evidenced DNA methylation/demethylation events in larvae and pupae, and significant differences in histone methylation and phosphorylation between newly emerged adult queens and workers. The epigenetic dynamics seen in this stingless bee species represent a new facet in the caste determination process in *Melipona* bees and suggest a possible mechanism that is likely to link a genotype component to the larval diet and adult social behavior of these bees.

## Introduction

Studies on caste determination in bees have largely benefited from the availability of a well annotated genome sequence from *Apis mellifera* (The Honey Bee Genome Sequencing Consortium, 2006). This directed the focus to a key issue, the role and function of nutrient sensing pathways as connectors between the differential nutrition of the queen and worker larvae and the endocrine signals related to differential gene expression (Hartfelder *et al*., 2015). In comparison, frustratingly little progress has been made for stingless bees with respect to understanding how caste is determined since this question has last been comprehensively reviewed (Hartfelder *et al*., 2006).

While in most highly eusocial bees, wasps and ants, the genome of a single individual is capable of being nutritionally driven to express one of the alternative phenotypes (typically the queen and worker morph, but also additional worker morphs in ants), caste fate in the genus *Melipona*, as well as in certain ants, such as *Pogonomyrmex*, is biased by the genotype (for a review see Corona *et al*., 2016). In stingless bees of the genus *Melipona*, queens, workers and males emerge from brood cells of the same size with no differences in the quantity of the diet supplied to the larvae (Kerr, 1946, 1950; Beig *et al*., 1985). The still most accepted mechanistic hypothesis of caste determination in *Melipona* is the interaction of genetic and environmental factors that jointly influence juvenile hormone (JH) biosynthesis (Velthuis, 1976; Bonetti *et al*., 1995). According to this hypothesis, double heterozygosity at two not yet identified loci, coupled with an adequate food supply would result in high levels of JH production, and under optimal colony conditions, this predicted mechanism could explain the observed 3:1 worker to queen ratio in newly emerged female brood (Kerr and Nielsen, 1966).

The two loci/two alleles hypothesis with classic Mendelian segregation as the genetic basis of caste determination in the genus *Melipona* (Kerr, 1950) has a long history of controversy (see Hartfelder *et al*., 2006), especially because most of the issues raised fall into the debate of developmental (mechanistic) factors *vs*. evolutionary (ultimate) causes. In *Melipona*, neither the queen nor the brood-tending workers have apparently control over the caste fate of the brood, which is raised in cells of equal size and practically on the same quantity of larval food, in complete contrast to the nutritional caste determination in the honey bee (for a recent review see Hartfelder *et al*., 2015). Hence, on theoretical grounds, it was proposed that the brood should have the potential to self-determine its proper caste fate, and modeling of the inclusive fitness of *Melipona* females actually predicted an optimum queen-emergence frequency of 20% in the female brood of a colony headed by a single-mated queen (Wenseleers *et al*., 2003; Wenseleers and Ratnieks, 2004). Clearly, this is very close to the ratio observed and postulated under the Mendelian genetics two loci/two alleles hypothesis. Nonetheless, there is now evidence that a specific compound in larval food, geraniol, may have a queen-fate inducing effect in *Melipona* bees (Jarau *et al*., 2010).

The discussion on the mechanistic and, especially so, the molecular basis of caste determination in the stingless bee genus *Melipona* has recently gained important insights from the molecular mechanisms underlying sex determination in the honey bee. The *feminizer* (*fem*) gene, which is sex-specifically spliced in bees (Biewer *et al*., 2015), is not only sex-specifically spliced during embryonic development in relation to sex determination, but was also evidenced to be sex- and caste-specifically expressed during late larval development in *Melipona interrupta* (Brito *et al*., 2015). Furthermore, these same authors showed that *fem* is also regulated by JH during the larval stage critical for caste differentiation. Though unlikely to be one of the actual two loci for genetic caste determination in *Melipona* bees, as proposed by Kerr (1950), the *fem* gene is nonetheless the first *bona fide* candidate gene involved in caste development. Nonetheless, the integration between JH and the gene regulatory networks that drive differential gene expression related to caste development in stingless bees is still an open question.

Recently, the *Melipona quadrifasciata* genome has been fully sequenced as part of a comparative genomic study on 10 bee species (Kapheim *et al*., 2015). One remarkable conclusion from this study was, that as sociality gets more complex, the bees present a higher number of potential methylation sites on gene bodies. Hence, the increase in CpG sites in the bee genomes concurrent with the social level should be of broad significance, especially so in the light of the functional importance of DNA methylation in honey bee caste development (Kucharski *et al*., 2008), and could present an important factor within the genetic determination of caste in the stingless bee genus *Melipona*.

DNA methylation and post-translational histone modifications have been extensively investigated in chromatin reorganization of many vertebrates (Gabor Miklos and Maleszka, 2011). However, for invertebrates, epigenetic modifications and their functional roles in the control of gene expression are still controversial issues. This is largely so because among the two main invertebrate model systems the nematode *Caenorhabditis elegans* does not present any DNA methylation at all (Gabor Miklos and Maleszka, 2011), and the genome of the fruit fly, *Drosophila melanogaster*, only encodes a DNA methyltransferase 2 homolog, which has no *in vitro* activity and is not capable of methylating CpG dinucleotides (Tweedie *et al*., 1999; Lyko *et al*., 2000; Kunert *et al*., 2003). Furthermore, a methylomics study on the mosquito *Aedes aegypti* could not detect a defined DNA methylation profile, and RNA bisulfite sequencing revealed that methylation in tRNAs may actually be implicated in dengue virus replication (Falckenhayn *et al*., 2016). In contrast, the red flower beetle, *Tribolium castaneum*, shows DNA methylation (Feliciello *et al*., 2013), and its genome has the potential to encode at least two DNA methyltransferases (DNMTs), a DNMT1 and a DNMT2 (Tribolium Genome Sequencing Consortium *et al*., 2008).

Within the order Hymenoptera, DNA methylation has become an important topic once a complete set of DNA methyltransferases (DNMTs) was evidenced in the sequenced genome of the honey bee (Wang *et al*., 2006), and the functional importance of DNMT3 in caste phenotype determination was convincingly demonstrated by Kucharski *et al*. (2006). Subsequent studies then revealed extensive differential DNA methylation in honey bees related to caste development (Foret *et al*., 2012) and learning and memory formation (Lockett *et al*., 2010). Furthermore, a direct association between differential DNA methylation and alternative splicing was put in evidence (Foret *et al*., 2012; Li-Byarlay *et al*., 2013). Several histone post-translation modifications have also been identified in honey bees (Dickman *et al*., 2013). Similar findings on epigenetic chromatin modification, especially DNA methylation, was also reported for several species of ants (Bonasio *et al*., 2012; Bonasio, 2014; Alvarado *et al*., 2015; Glastad *et al*., 2015), leading to infer that DNA methylation is associated with and plays an important role in caste and life cycle regulation across social Hymenoptera (Weiner *et al*., 2013).

Here we hypothesize that an epigenetically modulated chromatin state may affect caste fate in *Melipona* bees, and we investigated this question in a pilot study on *Melipona scutellaris*. Our goals were to obtain evidence for the presence of an active DNA methylation system and post-translational histone H3 tail modifications in these bees, and to see how the resultant epigenetic states may be differentially modulated and related to sex and caste development and to adult behavioral states.

## Materials and Methods

### Bees

*Melipona scutellaris* larvae, pupae, newly emerged queens, males and workers, as well as adult workers performing different roles (nurses and foragers) were collected from colonies maintained in the meliponary of the Federal University of Uberlândia. The classification of developmental stages used here is described in Dias *et al*. (2001) and Vieira *et al*. (2008)._After sampling, the bees were immediately frozen in liquid nitrogen and stored at -80 °C until use.

### DNA extraction and methylated cytidine quantification

We quantified the levels of global DNA methylation using an ELISA-based assay following the protocols established for aphids (Ayyanath *et al*., 2014) and the honey bee (Shi *et al*., 2011). This methodology has been successfully employed to identify DNA methylation differences without requiring a known genome sequence (Olkhov-Mitsel and Bapat, 2012). Briefly, genomic DNA from individual bees of each developmental stage was extracted by the CTAB method (Chen *et al*., 2010) and quantified using a NanoDrop Spectrophotometer (ND-1000, Thermo Scientific). The content of methylated cytidine bases was assessed by means of the EIA DNA Methylation kit (Cayman Chemicals, Ann Arbor, MI, USA). Two dilutions were used to calculate the respective OD values by regression against a standard curve prepared as recommended by the manufacturer. Since the larval OD values exceeded the linear range of the standard curve, their DNA methylation content is reported semiquantitatively only, as highly methylated DNA. Each age group or sample type is represented by three individual bees as biological replicates.

### Histone acid extraction and western blot analysis

Histone post-translational modifications were investigated by western blot analysis of acid extracted histones from five individual bees of each sample type. The acid extraction followed the Abcam protocol (http://www.abcam.com/protocols/histone-extraction-protocol-for-western-blot). Briefly, the bees were macerated in liquid nitrogen and incubated for 10 min on ice with Triton extraction buffer (TEB) consisting of PBS containing 0.5% Triton X100 (v/v), 2 mM phenylmethylsulfonyl fluoride (PMSF) and 0.02% (w/v) sodium azide (NaN_3_). After centrifugation at 2,000 x *g* at 4 °C the supernatant was discarded and the nuclear pellet washed in 1 mL of TEB. Following a second centrifugation step, the pellet was resuspended in 0.2 N HCl overnight for acid extraction of histones at 4 °C. The supernatant containing acid-soluble proteins, including acidic histones, was dialyzed twice against acetic acid for 1 h and 3 times against distilled water (1 h, 3 h and overnight) according to Di Paola *et al*. (2012). The dialyses were performed with 1000 MWCO Spectra/Por^TM^ membranes (Spectrum Laboratories, Rancho Dominguez, CA). The protein concentrations of the histone extracts were determined by Bradford assay, following the manufacturer's protocol (Bio-Rad, Hercules, CA, USA).

Fifty-two micrograms of histone protein extract were separated by 17.5% SDS-PAGE followed by electrophoretic transfer onto Hybond^TM^ ECL 0.2 μM nitrocellulose membranes (Amersham, Bucks, UK) overnight at 80 mA. The membranes were blocked with 3% non-fat milk diluted in PBS for 1 h, incubated with primary antibodies for 3 h, washed three times with PBS, and incubated with secondary antibodies for 1 h. Subsequently, the membranes were washed three times with PBS before incubation with ECL Western Blot Detection Reagent (Amersham). The immunoreactive bands were detected with a GBX detection reagent (Kodak) and Hyperfilm^TM^ ECL (Amersham). The following antibodies were used: anti-H3T3-P (Cell Signaling Technology, Danvers, MA), anti-H3K4-Me (Cell Signaling), anti-H2B (Abcam, Cambridge, UK) and HRP-conjugated anti-rabbit IgG (Sigma-Aldrich, Saint Louis, MO) in appropriate replicates and using the manufacturers suggestions for ideal dilutions. Band intensity was quantified using ImageJ^®^ software 1.47v. The data were normalized by the control band intensity. The sample analyses were replicated three times.

### Statistical analysis

For the DNA methylation assays, the average differences between larval stages, caste and sex of pupae and adults were analyzed by a Kruskal-Wallis test followed by Dunn's post-hoc tests. A one-tailed Mann-Whitney test was used to compare average differences between pupae and adult individuals. For the histone modification analyses, the western blot image data were confirmed for normality by a Kolmogorov-Smirnov test before running a two-tailed unpaired Student's *t*-test. All statistical analyses were performed with GraphPad Prism 5 (GraphPad Software Inv., La Jolla, CA, USA), with a significance value set at p < 0.05.

## Results and Discussion

### Global DNA methylation levels in *Melipona scutellaris* sexes and castes

The content of methylated DNA of specimens of the stingless bee *M. scutellaris* was calculated for pupae and adult bees of both sexes and castes. For larvae, only a general level of DNA methylation could be assessed, because it is not possible to distinguish sex and caste in larval stages (Amaral *et al*., 2010).

**Figure 1:**
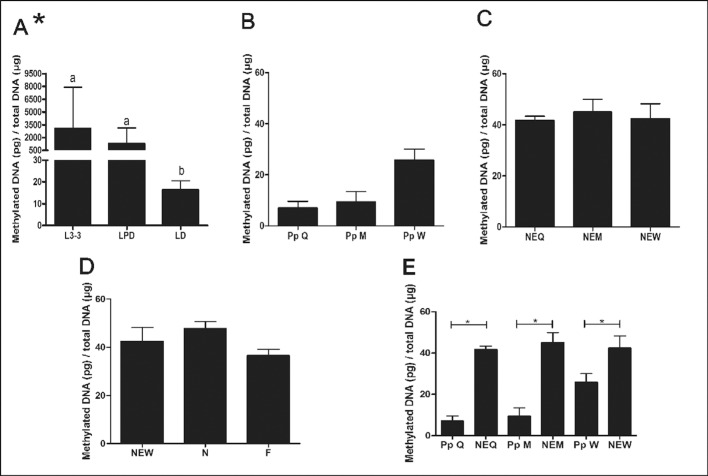
Quantification of methylated cytidine content in the genomic DNA of *Melipona scutellaris* analyzed by ELISA. (A) Quantification of methylated DNA of larvae, (B) pupae, (C) newly emerged individuals, and (D) adult workers. In (E) the methylated cytidine content is shown for queens, workers and males, contrasting pupae against newly emerged individuals. L3-3 = larva of the third stage in the third instar; LPD = pre-defecating larva; LD = defecating larva; PpQ = pink-eyed queen pupa; PpM = pink-eyed male pupa; PpW = pink-eyed worker pupa; NEQ = newly emerged queen; NEM = newly emerged male; NEW = newly emerged worker; N = nurse; F = forager. The graphs show means ± SEM (n = 3) for methylated DNA (pg) per genomic DNA (μg), * semiquantitative estimates since the larval genome is hypermethylated,. Statistical analysis: (A-D) Kruskal-Wallis with a post-hoc Dunn's test, p < 0.05; (E) one-tailed Mann-Whitney test, p < 0.05.

The DNA of larvae of *Melipona scutellaris* turned out to be strongly hypermethylated (Figure 1A), followed by a fast demethylation event during the larval-pupal transition. Worker pupae had a threefold higher methylation content in their DNA when compared to queens and males of the same developmental stage (Figure 1B), and when specifically comparing the two female castes, the global DNA methylation content in pupae of workers was significantly different from that in queens (unpaired *t*-test, p < 0.05). Thus, the differential DNA methylation content may play a role in the expression of the caste and sex phenotypes of *M. scutellaris*, similar to what has been denoted in honey bees. Analyses of the methylome of honey bee larvae revealed differentially methylated genes related to the regulation of JH biosynthesis and also showed that splicing is regulated by DNA methylation (Foret *et al*., 2012; Li-Byarlay *et al*., 2013). In *M. scutellaris*, the methylation content increased significantly, almost twofold, as the pupae developed into adult bees, but there were no significant differences among castes or among workers of different age or function in the colonies (foragers and nurses) (Figure 1C,D). Interestingly, similar results were seen in queens and workers of *A. mellifera*, where no differentially methylated regions were found in newly emerged bees (Herb *et al*., 2012). However, approximately 560 genes in brains of mature adult and 2,390 genes in larval honey bees were identified to be differentially methylated when comparing queens and workers (Lyko *et al*., 2010; Foret *et al*., 2012; Herb *et al*., 2012), suggesting that even without differences in global DNA methylation, specific regions may be differentially methylated also between the castes and sexes of *M. scutellaris*. Thus, the transition from nurse to forager of *M. scutellaris* workers may be associated with gene-specific methylation, as observed in honey bees, but this is a hypothesis that remains to be tested by the analysis of actual DNA methylation patterns through bisulfite sequencing.

Gains and losses of DNA methylation have been related to developmental plasticity in the parasitic wasp *Nasonia vitripennis* (Zwier *et al*., 2012) and also in the honey bee (Drewell *et al*., 2014), and our data reported here for *M. scutellaris* suggest that DNA methylation may be of general importance during the development of this social insect (Figure 1). In mammals, DNMT3 is responsible for *de novo* methylation activity and is a key driver of global DNA methylation reprogramming (Bestor, 2000), and for *A. mellifera*, DNMT3 has been convincingly shown to play a critical role in caste differentiation (Kucharski *et al*., 2008). Hence, similar differences in the enzymatic activity of DNMT3 could be responsible for *de novo* DNA methylation in *M. scutellaris*, where global DNA methylation levels drop during the last larval instar (Figure 1A) and then increase with apparently differential dynamics during the pupal stages, before attaining similar levels in the newly emerged adult queens, workers and males (Figure 1B,C).

The variation in global DNA methylation levels during the last larval instar and pupal development presents an interesting potential connection with the divergent JH and ecdysteroid titers seen in caste development of stingless bees (Hartfelder and Rembold, 1991, Hartfelder *et al*., 2006), and alternative splicing of a key gene in the JH biosynthesis pathway (Vieira *et al*., 2008) and of the *fem* gene (Brito *et al*., 2015) in the genus *Melipona*. Our results, thus, suggest that epigenetic modifications may represent a link between genotype and environment in the development of the queen or worker phenotypes of *Melipona* bees, and this hypothesis is supported by the epigenetics data shown in Figure 1 and by previous results in the literature on caste ratios and JH signaling in these bees (Kerr, 1946, 1950, Bonetti *et al*., 1995).

The presence of DNA methylation on CpG sites in stingless bees has been predicted from the genome sequence of *M. quadrifasciata*, and we provide here clear evidence for both DNA methylation and histone modification in *M. scutellaris*, in relation to sex and caste development and the adult life cycle. The similarity of these highly eusocial bees to honey bees in the degree of sociality should, thus, stimulate further studies on the subfamily Meliponini. Both stingless bees and honey bees are highly eusocial, but they belong to different branches within the clade of corbiculate bees (Cardinal *et al*., 2010; Hedtke *et al*., 2013). Furthermore, the Meliponini, with hundreds of species distributed across more than 50 genera (Camargo and Pedro, 2007; Rasmussen and Cameron, 2010) are much more diverse than the honey bees (Apini), represented only by a single genus, *Apis*, that comprises just seven extant species (Engel, 1999). With respect to the *Melipona* species mentioned in this study it is worthy of note that *Melipona scutellaris* Latreille 1811 belongs to the subgenus *Michmelia*, *Melipona interrupta* Latreille 1811 to the subgenus *Melikerria*, and *Melipona quadrifasciata* Lepeletier 1836 to the subgenus *Eomelipona*. *Eomelipona* and *Michmelia* are sister groups that became separated approximately 16 mya, whereas *Melikerria* diverged earlier from these two, approximately 20 mya (Rasmussen and Cameron, 2010). Nonetheless, like all *Melipona* species, they have in common a genetic caste determination mechanism. In several species of the genus *Melipona*, as well as in other Meliponini, a role for juvenile hormone in queen phenotype induction has been put in evidence, but epigenetic aspects have, so far, only been addressed in *M. scutellaris*.

### Histone modification in *Melipona scutellaris* sexes and castes

**Figure 2:**
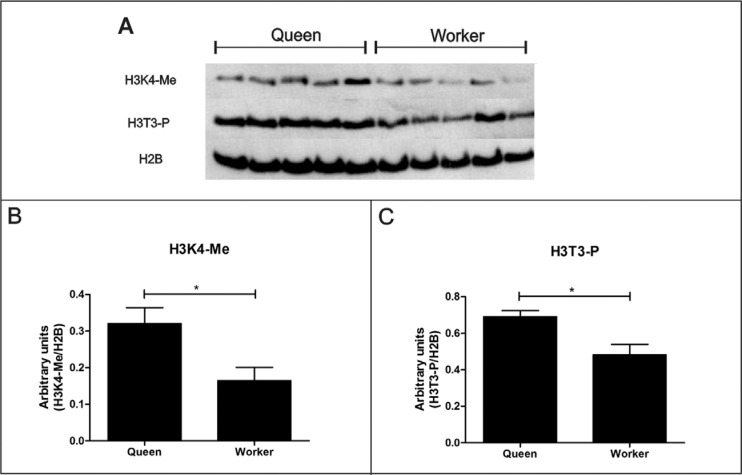
Histone post-translational modifications in newly emerged queens and workers of *Melipona scutellaris*. (A) Western blot showing mono-methylation of lysine 4 of histone H3 (H3K4-Me) and phosphorylation of threonine 3 of histone 3 (H3T3-P); histone H2B (H2B) was used as loading control. (B) Quantification of H3K4-Me bands and (C) Quantification of H3T3-P bands by ImageJ^®^ software. The y-axes of the graphs represent arbitrary units for intensity of H3K4-Me and H3T3-P bands normalized by H2B band intensity. Bars shown in B and C represent means ± SEM. Statistical analysis was done by one-tailed, unpaired Student's *t*-tests, p < 0.001, n = 5.

Here we tested the presence of post-translational modifications on histone H3 tails. Two distinct, well-established histone post-translational modifications, phosphorylation of threonine 3 on histone H3 (H3T3-P) and mono-methylation of lysine 4 on histone H3 (H3K4-Me), were semiquantitatively analyzed from western blots. We could show that queens have higher levels of both types of histone post-translational modifications when compared to workers of the same age class (Figure 2A), with significant differences in optical densities of the bands (Figure 2B,C; unpaired Student's *t*-test t, p < 0.05).

The two types of histone modifications are involved in gene regulation, but they act in distinct ways. H3T3-P is present mainly on heterochromatin domains that are highly represented in mitotically dividing cells, where they were shown to repress gene expression in mammalian cell lines (Varier *et al*., 2010). In contrast, H3K4-Me is known to act on regulatory sites of the mammalian genome, such as active enhancers (Wu and Ng, 2011; Zhou *et al*., 2011).

## Conclusions

**Figure 3:**
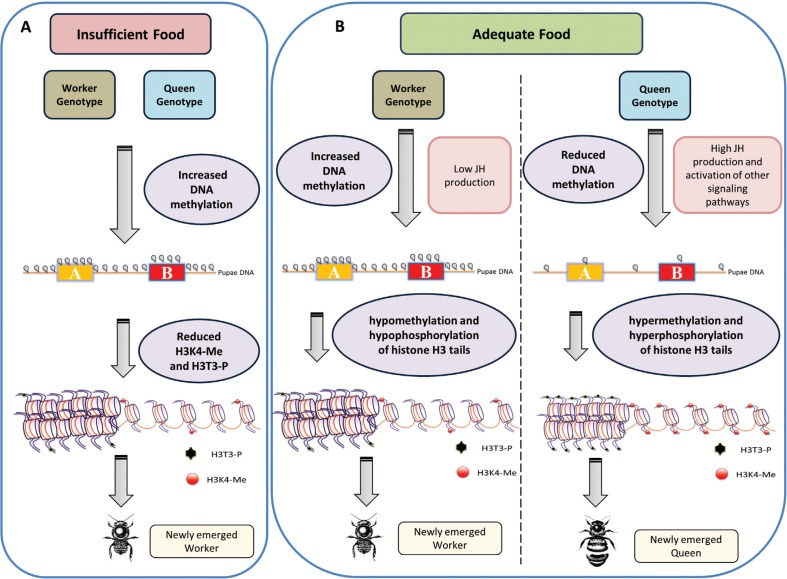
A schematic model on the interaction of caste genotype (heterozygosity at the two caste loci in queens, or homozygosity for at least one locus in workers), juvenile hormone (JH) production and epigenetic modifications in *Melipona scutellaris* caste differentiation. (A) Caste differentiation under conditions of insufficient food conditions provided to the larvae. In this case, the genotype bias becomes irrelevant, JH production is low and is associated with DNA hypermethylation during the pupal stage. Subsequently, on adult emergence, DNA is hypomethylated and there is hypophosphorylation of histone H3 tails. (B) Under adequate food conditions the caste genotype comes to play a role, and JH production and possibly other signaling pathways become activated in the queen larvae. The queen genome becomes hypomethylated during the larval-pupal transition, followed by hyperphosphorylation and hypermethylation of histone H3 tails when the adult queens emerge. In contrast, in worker genotype larvae, JH production is not activated and an increase in DNA methylation occurs at the larval-pupal transition, followed by hypomethylation and hypophosphorylation of histone H3 tails at the end of preimaginal development. In the adult worker bees, the differences in global methylation then likely favor gene activation related to division of labor (nurses and foragers). Yellow and red boxes with the letters A and B represent the two loci proposed in Kerr's model (Kerr, 1950).

Our results provide evidence for a composite epigenetic system in *Melipona* bees, where DNA methylation may control events during the preimaginal developmental stages, while histone post-translational modifications are likely to fine-tune gene expression during the adult life cycle of the bees. In a schematic model (Figure 3) we now propose that in larvae reared under insufficient food conditions (Figure 3A), a global cytosine methylation associated with low levels of JH production should result in worker development, independent of the individual's genotype condition. The newly emerged workers would then exhibit DNA hypomethylation and hypophosphorylation of histone H3 tails, leading to subsequent differential gene expression related to the performance of worker tasks (Figure 3A). With adequate feeding (Figure 3B), the queen genotype larvae/pupae show reduced DNA methylation associated with elevated JH production and likely, activation of other signaling pathways, such as the insulin/insulin-like and TOR pathways. The newly emerged queens would then exhibit hypermethylation and hyperphosphorylation of histone H3 tails, resulting in the fine tuning of queen-specific gene expression. If larvae have a worker genotype (homozygosity for at least one of the two predicted loci), the adequate feeding conditions alone should not be sufficient to achieve a hypomethylated state of the larval/pupal epigenome, and associated with low JH production, this would result in worker phenotype individuals (Figure 3B, left side).

We finally emphasize the importance of the pioneering studies of Prof. Dr. Warwick E. Kerr, which led him to conclude that caste determination in these stingless bees has a strong genetic component. As a leading geneticist of his time, he formulated this as a two loci/two alleles hypothesis with Mendelian segregation to explain the observed 3:1 segregation of workers and queens in the emerging brood. This eminent Brazilian researcher has brought the stingless bees into the limelight of sociobiology research and paved the way for subsequent studies on the biology of this diverse group of tropical highly eusocial bees, which are important pollinators for crops and native flora.
